# Effects of combined rAAV-mediated TGF-β and *sox9* gene transfer and overexpression on the metabolic and chondrogenic activities in human bone marrow aspirates

**DOI:** 10.1186/s40634-017-0077-5

**Published:** 2017-02-07

**Authors:** Ke Tao, Ana Rey-Rico, Janina Frisch, Jagadeesh Kumar Venkatesan, Gertrud Schmitt, Henning Madry, Jianhao Lin, Magali Cucchiarini

**Affiliations:** 1 Institute of Arthritis, Peking University People’s Hospital, No. 11 Xizhimen Nan Road, Xicheng District, Beijing, 100044 People’s Republic of China; 2 Center of Experimental Orthopaedics, Saarland University Medical Center, Kirrbergerstr. Bldg 37, D-66421 Homburg/Saar, Germany; 3 Department of Orthopaedic Surgery, Saarland University Medical Center, Homburg/Saar, Germany

**Keywords:** Human bone marrow aspirates, Recombinant adeno-associated virus, Combined gene transfer, Transforming growth factor beta, SOX9, Chondrogenesis

## Abstract

**Background:**

Transplantation of genetically modified bone marrow concentrates is an attractive approach to conveniently activate the chondrogenic differentiation processes as a means to improve the intrinsic repair capacities of damaged articular cartilage.

**Methods:**

Human bone marrow aspirates were co-transduced with recombinant adeno-associated virus (rAAV) vectors to overexpress the pleiotropic transformation growth factor beta (TGF-β) and the cartilage-specific transcription factor *sox9* as a means to enhance the chondroreparative processes in conditions of specific lineage differentiation.

**Results:**

Successful TGF-β/*sox9* combined gene transfer and overexpression via rAAV was achieved in chondrogenically induced human bone marrow aspirates for up to 21 days, the longest time point evaluated, leading to increased proliferation, matrix synthesis, and chondrogenic differentiation relative to control treatments (reporter *lacZ* treatment, absence of vector application) especially when co-applying the candidate vectors at the highest vector doses tested. Optimal co-administration of TGF-β with *sox9* also advantageously reduced hypertrophic differentiation in the aspirates.

**Conclusions:**

These findings report the possibility of directly modifying bone marrow aspirates by combined therapeutic gene transfer as a potent and convenient future approach to improve the repair of articular cartilage lesions.

## Background

Articular cartilage over the surface of epiphysis of bone is a highly specialized connective tissue formed by chondrocytes, enabling almost smooth, frictionless movement between the articulating surfaces of diarthrodial joints. Due to its intrinsic limited potential for self-healing, damaged articular cartilage is not capable of restoring its original structure and functions and often degenerates towards osteoarthritis. Currently available therapeutic options in the clinics have increasingly focused on cell-based approaches including bone marrow stimulation techniques (microfracture, pridie drilling, abrasion) that promote the penetration of chondroregenerative cells from the subchondral bone marrow in sites of cartilage lesions (Madry et al. 2010). Yet, such procedures do not allow to reproduce the original cartilage, allowing only for the production of a fibrocartilaginous repair tissue made of type-I collagen and of poor mechanical quality, but not of the native, highly organized hyaline cartilage (proteoglycans, type-II collagen) capable of supporting joint loading and motion (Breinan et al. 2000; Frisbie et al. 2003; Madry et al. 2010; Orth et al. 2014).

While administration of human bone marrow-derived mesenchymal stem cells (hMSCs) has been already attempted in patients to activate the regenerative processes in focal cartilaginous lesions (Haleem et al. 2010; Kuroda et al. 2007; Nejadnik et al. 2010; Orth et al. 2014; Skowronski and Rutka 2013; Wakitani et al. 2004; Wakitani et al. 2007), the necessity of applying multiple steps to harvest the cells and propagate them over time in vitro has weakened its widespread clinical application. Application of bone marrow concentrates containing chondrogenically competent MSCs among other cell populations (hematopoietic cells, fibroblast-like cells) in a natural biochemical (growth factors) microenvironment instead may provide less demanding approaches for the goal of therapeutic transplantation in cartilage defects (Gigante et al. 2012; Kim et al. 2014; Slynarski et al. 2006) as also performed to treat femoral head necrosis and nonunion bone fractures (Hauzeur and Gangji 2010; Kon et al. 2012). Still, even though encouraging results have been obtained when implanting such samples in preclinical models and in patients, the current outcomes have not been consistently associated with the formation of a functional, hyaline-like repair tissue that fully and stably integrates with the surrounding, intact cartilage (Gigante et al. 2012; Ivkovic et al. 2010; Kim et al. 2014; Pascher et al. 2004; Slynarski et al. 2006).

In this regard, genetic modification of bone marrow concentrates by transfer of sequences coding for chondrogenic and/or chondroreparative factors prior to implantation in sites of cartilage injury might be a powerful tool to overcome such critical issues (Frisch et al. 2015; Johnstone et al. 2013). Various therapeutic sequences have been tested to achieve this goal, including the cartilage oligomeric matrix protein (COMP), bone morphogenetic proteins (BMPs), transforming growth factor beta (TGF-β), insulin-like growth factor I (IGF-I), basic fibroblast growth factor (FGF-2), the sex-determining region Y-type high-mobility group box (SOX) family of transcription factors, zinc finger protein 145 (ZNF145), Indian hedgehog (Ihh), Wnt11, and small interfering RNA (siRNA) against p53 and runt-related transcription factor 2 (Runx2) (Babister et al. 2008; Cucchiarini et al. 2009; Cucchiarini et al. 2011; Frisch et al. 2014; Haleem-Smith et al. 2012; Huang et al. 2010; Ikeda et al. 2004; Jeon et al. 2012; Kim and Im 2011; Lee et al. 2011; Liu et al. 2011; Liu et al. 2013; Liu et al. 2014; Liu et al. 2015; Neumann et al. 2013; Pagnotto et al. 2007; Steinert et al. 2009; Steinert et al. 2012; Tao et al. 2016; Venkatesan et al. 2012). Interestingly, previous reports from diverse groups, among which ours, demonstrated that multiple therapeutic gene transfer might be necessary to promote optimal reparative activities in various cells of the musculoskeletal system (articular chondrocytes, MSCs) (Cucchiarini et al. 2009; Ikeda et al. 2004; Kim and Im 2011; Liu et al. 2013; Liu et al. 2015; Steinert et al. 2009; Tao et al. 2016).

In light of our work showing the therapeutic benefits of overexpressing TGF-β simultaneously with *sox9* in isolated human MSCs (Tao et al. 2016) and independently in human bone marrow aspirates (Frisch et al. 2016; Rey-Rico et al. 2015) without any interference of independent vectors in dual *versus* single gene transfer, we tested here the possibility of co-delivering these two highly chondrogenic factors to further enhance the repair processes in primary human bone marrow aspirates. We specifically focused on gene transfer using the clinically adapted recombinant adeno-associated virus (rAAV) vectors that can transduce MSCs at very high efficiencies (up to 100%) and over extended periods of time (at least 3 weeks) without altering their differentiation potential (Cucchiarini et al. 2009; Cucchiarini et al. 2011; Frisch et al. 2014; Lee et al. 2011; Pagnotto et al. 2007; Tao et al. 2016; Venkatesan et al. 2012). Of further note, transduction via rAAV does not raise viral interference, allowing for concomitant administration of independent vectors in their targets (Cucchiarini et al. 2009).

For the first time to our best knowledge, we provide evidence that successful, prolonged co-overexpression of TGF-β and *sox9* using this vector class synergically enhances the levels of proliferation, biosynthesis, and chondrogenesis in human bone marrow concentrates relative to control conditions (reporter *lacZ* treatment, absence of vector application) while delaying undesirable hypertrophic and osteogenic differentiation. These observations support the concept of modifying bone marrow aspirates by multiple rAAV vectors as a promising approach for future implantation procedures in articular cartilage defects in vivo.

## Methods

### Chemicals and reagents

All reagents were purchased at Sigma (Munich, Germany) unless otherwise indicated. The dimethylmethylene blue dye was from Serva (Heidelberg, Germany). Recombinant TGF-β3 was from Peprotech (Hamburg, Germany). The antibodies used for immunohistochemical analyses were as follows: the anti-β-galactosidase (β-gal) (GAL-13) and anti-type-X collagen (COL-10) antibodies from Sigma, the anti-TGF-β (V), anti-SOX9 (C-20), and anti-FLAG tag (BioM2) antibodies from Santa Cruz Biotechnology (Heidelberg, Germany), the anti-type-I collagen (COL-1) antibody from Abcam (Cambridge, UK), and the anti-type-II collagen (II-II6B3, NIH Hybridoma Bank, University of Iowa, Ames, USA) antibody from Acris (Hiddenhausen, Germany). Biotinylated secondary antibodies and the ABC reagent were purchased at Vector Laboratories (Alexis Deutschland GmbH, Grünberg, Germany). The TGF-β enzyme-linked immunosorbent assay (active hTGF-β1 Quantikine ELISA) was from R&D Systems (Wiesbaden, Germany).

### Human bone marrow aspirates

Human bone marrow aspirates (~15 ml; 1.4 ± 0.4 × 10^9^ cells/ml) were obtained from the distal femurs of osteoarthritic patients undergoing total knee arthroplasty (*n* = 8, age 69 ± 11 years) (Frisch et al. 2016; Rey-Rico et al. 2015). All patients provided informed consent before inclusion in the study and all procedures were in accordance with the Helsinki Declaration. The study was approved by the Ethics Committee of the Saarland Physicians Council (Application 06/08).

### Plasmids and rAAV vectors

The parental AAV-2 genomic clone pSSV9 was used to create all constructs applied in this study (Samulski et al. 1987; Samulski et al. 1989). rAAV-*lacZ* carries the *lacZ* gene for *E. coli* β-galactosidase under the control of the cytomegalovirus immediate-early (CMV-IE) promoter. rAAV-hTGF-β carries a 1.2-kb human transforming growth factor beta 1 (hTGF-β1, active form) cDNA fragment and rAAV-FLAG-h*sox9* a 1.7-kb FLAG-tagged human *sox9* (h*sox9*) cDNA, both cloned in rAAV-*lacZ* in place of *lacZ* (Cucchiarini et al. 2009; Cucchiarini et al. 2011; Frisch et al. 2014; Frisch et al. 2016; Rey-Rico et al. 2015; Tao et al. 2016; Venkatesan et al. 2012). rAAV were packaged as conventional (not self-complementary) vectors using the 293 adenovirus-transformed embryonic kidney cell line. Adenovirus 5 was used to provide helper functions in combination with the pAd8 helper plasmid as previously described (Cucchiarini et al. 2009; Cucchiarini et al. 2011; Frisch et al. 2014; Frisch et al. 2016; Rey-Rico et al. 2015; Tao et al. 2016; Venkatesan et al. 2012). The vectors were purified, dialyzed, and titrated by real-time PCR (Cucchiarini et al. 2009; Cucchiarini et al. 2011; Frisch et al. 2014; Frisch et al. 2016; Rey-Rico et al. 2015; Tao et al. 2016; Venkatesan et al. 2012), averaging 10^10^ transgene copies/ml (ratio virus particles to functional vectors = 500/1).

### rAAV-mediated gene transfer

Aspirates were aliquoted in standard tissue culture plastic 96-well plates (100 μl of aspirate/well) and immediately transduced with the rAAV vectors (rAAV-*lacZ*: 20 or 40 μl) or co-transduced (rAAV-hTGF-β/rAAV-FLAG-h*sox9*: 10 or 20 μl each vector) with each aliquot (8 × 10^5^ functional recombinant viral particles, MOI = 10 ± 3) (Frisch et al. 2016; Rey-Rico et al. 2015), while aspirates were added only 40 μl DMEM medium as the negative control group. A volume of 60 μl of chondrogenic medium (4.5 g/l DMEM high glucose, 100 U/ml penicillin and 100 μl/ml streptomycin, 6.25 μg/ml insulin, 6.25 μg/ml transferrin, 6.25 μg/ml selenious acid, 5.35 μg/ml linoleic acid, 1.25 μg/ml BSA, 1 mM sodium pyruvate, 37.5 μg/ml ascorbate 2-phosphate, 10^−7^ M dexamethasone, 10 ng/ml TGF-β3) was then added per aspirate with 250 μl of the abovementioned medium change performed once per week for MSC chondrogenesis for up to 21 days (Barry et al. 2001; Frisch et al. 2016; Johnstone et al. 1998; Rey-Rico et al. 2015; Yoo et al. 1998). To avoid attachment on the bottom of the plates, the aspirates were carefully mixed after each medium change. For osteogenic and adipogenic differentiation, the aspirates were transduced using similar rAAV-mediated gene transfer conditions as those described above and then induced either toward osteogenic differentiation using the StemPro Osteogenesis Differentiation kit or adipogenic differentiation using the StemPro Adipogenesis Differentiation kit (Life Technologies GmbH, Darmstadt, Germany) (Frisch et al. 2016).

### Detection of transgene expression

To assess TGF-β secretion, 30 μl of culture supernatant were collected at the denoted time points 24 h after medium change and centrifuged to remove debris and TGF-β production was measured by ELISA as previously described (Frisch et al. 2016). Quantitative measurements were performed on a GENios spectrophotometer/fluorometer (Tecan, Crailsheim, Germany). Moreover, after 21 days, the aspirates were collected by centrifugation at 1.500 rpm/min for 5 min to form pellet and subsequently fixed in 4% formalin, dehydrated in graded alcohols, embedded in paraffin, and sectioned (3 μm). Transgene expression (*lacZ*, TGF-β, *sox9*, FLAG) was also assessed by immunohistochemical analyses using specific primary antibodies, biotinylated secondary antibodies, and the ABC method with diaminobenzidine as the chromogen (Cucchiarini et al. 2009; Cucchiarini et al. 2011; Frisch et al. 2014; Frisch et al. 2016; Rey-Rico et al. 2015; Tao et al. 2016; Venkatesan et al. 2012). To control for secondary immunoglobulins, the samples were processed with omission of the primary antibody. Samples were examined under light microscopy (Olympus BX 45) (Olympus, Hamburg, Germany).

### Histological, immunocytochemical, and immunohistochemical analyses

Aspirates were collected and centrifuged to form pellet, fixed in 4% formalin, dehydrated in graded alcohols, embedded in paraffin, and sectioned (3 μm). Sections were stained with hematoxylin eosin (H&E) for cellularity, toluidine blue for matrix proteoglycans, and alizarin red for matrix mineralization (Cucchiarini et al. 2009; Cucchiarini et al. 2011; Frisch et al. 2014; Frisch et al. 2016; Rey-Rico et al. 2015; Tao et al. 2016; Venkatesan et al. 2012). Immunohistochemical analyses were performed to monitor the expression of type-II, −I, and -X collagen using specific primary antibodies, biotinylated secondary antibodies, and the ABC method with diaminobenzidine as the chromogen (Cucchiarini et al. 2009; Cucchiarini et al. 2011; Frisch et al. 2014; Frisch et al. 2016; Rey-Rico et al. 2015; Tao et al. 2016; Venkatesan et al. 2012). To control for secondary immunoglobulins, sections were processed with omission of the primary antibody. Samples were examined under light microscopy (Olympus BX 45).

### Morphometric analyses

The levels of cells expressing the transgene (% of β-gal^+^ cells), the cell densities on H&E-stained sections, the densities of toluidine blue and alizarin red staining and those of type-II, −I, and -X collagen, TGF-β, and SOX9 immunostaining were monitored at three random standardized sites or with 10 serial histological and immunohistochemical sections for each parameter, test and replicate condition using the SIS analySIS program (Olympus), Adobe Photoshop (Adobe Systems, Unterschleissheim, Germany), and Scion Image (Scion Corporation, Frederick, MD, USA) (Cucchiarini et al. 2009; Cucchiarini et al. 2011; Frisch et al. 2014; Frisch et al. 2016; Rey-Rico et al. 2015; Tao et al. 2016; Venkatesan et al. 2012). To evaluate β-gal expression, only cells strongly stained *versus* (faint) background DAB signal, i.e. in the absence of primary antibody, were considered as β-gal^+^. Regarding the measurements of the cell densities, H&E-stained sections from either *lacZ*-, TGF-β-, and *sox9*-treated aspirates were analyzed by counting cells per standardized area using a similar magnification (x40) for all types of samples in order to allow for strict comparison. To monitor the staining intensities, x20 images were first converted to inverted grayscale mode. Background DAB signal was adapted for comparable range of each type of immunostaining prior to evaluations. The total areas (mm^2^) covered with cells were next measured to identify the average gray value of the defined area. Data are given as mean intensity of staining or immunostaining per mm^2^ of total cell area.

### Biochemical assays

The aspirates were collected and digested with papain as previously described (Frisch et al. 2016). A fluorimetric assay using Hoechst 22358 was applied to determine the DNA contents and the proteoglycans were measured by binding to dimethylmethylene blue dye (Cucchiarini et al. 2009; Cucchiarini et al. 2011; Frisch et al. 2014; Frisch et al. 2016; Rey-Rico et al. 2015; Tao et al. 2016; Venkatesan et al. 2012). Total cellular proteins were monitored via protein assay (Pierce Thermo Scientific, Fisher Scientific, Schwerte, Germany) and subsequently used to normalize the biochemical data. All measurements were performed on a GENios spectrophotometer/fluorometer (Tecan).

### Real-time RT-PCR analyses

Total cellular RNA was extracted from all the aspirates at the denoted time points using TRIzol reagent (Ambion® Life Technologies) and the RNeasy Protect Mini Kit with an on-column RNase-free DNase treatment (Qiagen, Hilden, Germany) and RNA elution in 30 μl RNase-free water. 8 μl of eluate were applied to perform reverse transcription by using the 1^st^ Strand cDNA Synthesis kit for RT-PCR (AMV) (Roche Applied Science). Real-time PCR was carried out to amplify 2 μl of the cDNA product by using the Brilliant SYBR Green QPCR Master Mix (Stratagene, Agilent Technologies, Waldbronn, Germany) on an Mx3000P QPCR operator system (Stratagene) as follows: (95 °C, 10 min), amplification by 55 cycles (denaturation at 95 °C, 30 s; annealing at 55 °C, 1 min; extension at 72 °C, 30 s), denaturation (95 °C, 1 min), and final incubation (55 °C, 30 s) (Frisch et al. 2016). All primers were purchased at Invitrogen (Darmstadt, Germany): SOX9 (chondrogenic marker) (forward 5′-ACACACAGCTCACTCGACCTTG-3′; reverse 5′-GGGAATTCTGGTTGGTCCTCT-3′), aggrecan (ACAN) (chondrogenic marker) (forward 5′-GAGATGGAGGGTGAGGTC-3′; reverse 5′-ACGCTGCCTCGGGCTTC-3′), type-II collagen (COL2A1) (chondrogenic marker) (forward 5′-GGACTTTTCTCCCCTCTCT-3′; reverse 5′-GACCCGAAGGTCTTACAGGA-3′), type-I collagen (COL1A1) (osteogenic marker) (forward 5′-ACGTCCTGGTGAAGTTGGTC-3′; reverse 5′-ACCAGGGAAGCCTCTCTCTC-3′), type-X collagen (COL10A1) (marker of hypertrophy) (forward 5′-CCCTCTTGTTAGTGCCAACC-3′; reverse 5′-AGATTCCAGTCCTTGGGTCA-3′), and glyceraldehyde-3-phosphate dehydrogenase (GAPDH) (housekeeping gene and internal control) (forward 5′-GAAGGTGAAGGTCGGAGTC-3′; reverse 5′-GAAGATGGTGATGGGATTTC-3′) (all 150 nM final concentration) (Frisch et al. 2016). Water and non-reverse-transcribed mRNA were used as control conditions. To confirm the specificity of the products, melting curve analysis and agarose gel electrophoresis were performed. The threshold cycle (Ct) value for each amplified sample and each gene of interest was measured by using the MxPro QPCR software (Stratagene), and values were normalized to GAPDH expression by using the 2^-ΔΔCt^ method (Frisch et al. 2016).

### Statistical analyses

All conditions were performed in triplicates in three independent experiments for each patient and all patients were tested. Data are given as mean ± standard deviation (SD) of separate experiments. Statistical significance was assessed for any *p* value of less than 0.05 using the t-test and Mann–Whitney Rank Sum Test where appropriate.

## Results

### Effective and sustained TGF-β and *sox9* combined gene transfer and overexpression via rAAV in human bone marrow aspirates

Bone marrow aspirates were first transduced with the combination rAAV-hTGF-β/rAAV-FLAG-h*sox9* to evaluate the ability of rAAV to mediate direct co-overexpression of TGF-β and *sox9* over time in aspirates committed toward the chondrogenic differentiation compared with control conditions (rAAV-*lacZ* transduction, absence of vector treatment). As we previously reported that rAAV is capable of successfully expressing functional TGF-β and SOX9 factors separately in human bone marrow aspirates (Frisch et al. 2016; Rey-Rico et al. 2015), we did not include single gene treatments here as additional controls.

**Fig. 1:**
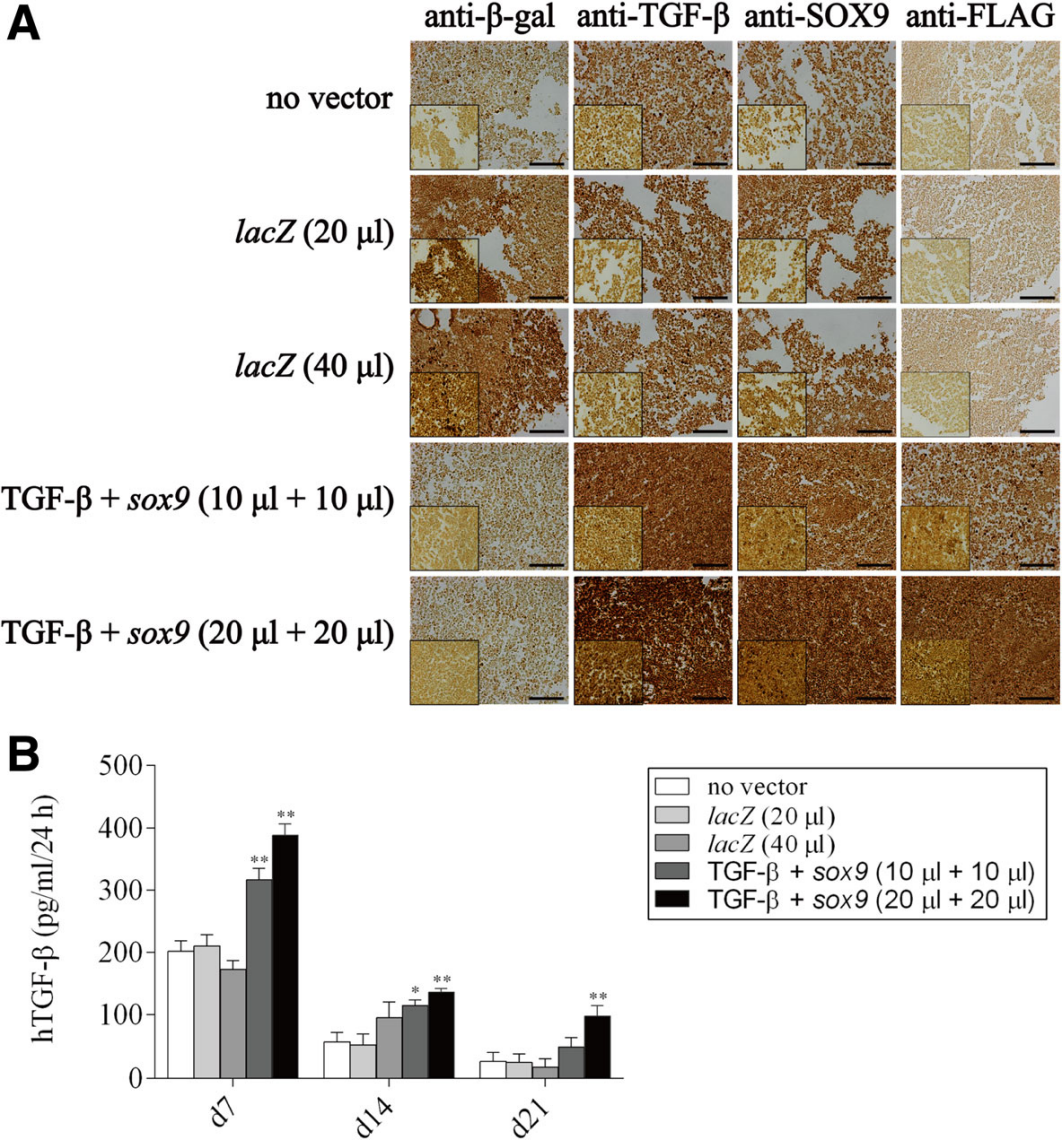
Detection of transgene expression in human bone marrow aspirates via rAAV-mediated gene transfer. Aspirates were co-transduced with rAAV-hTGF-β/rAAV-FLAG-h*sox9* (10 or 20 μl each vector), transduced with rAAV-*lacZ* (20 or 40 μl), or let untreated for over time (21 days) maintenance in chondrogenic medium as described in the Materials and Methods. The samples were processed after 21 days to detect (**a**) the expression of β-gal, TGF-β, SOX9, and the FLAG tag by immunohistochemistry (original magnification x20, bar = 100 μm, insets at x40, all representative data) and (**b**) the production of TGF-β by ELISA as described in the Materials and Methods. *,**Statistically significant compared with control groups (rAAV-*lacZ*, no vector condition) (*p ≤ 0.050, **p ≤ 0.010)

An evaluation of the % of the β-gal^+^ cells revealed higher transduction efficiencies in aspirates transduced with rAAV-*lacZ* after 21 days compared with the other conditions (2.7- to 3.7-fold difference at 20 or 40 μl vector, respectively, *p* ≤ 0.010) (Table 1) as estimated on immunohistochemical sections from aspirates (Fig. 1a). Strong, significant TGF-β expression was also observed in the aspirates after 21 days especially when rAAV-hTGF-β was provided to the aspirates as noted by immunohistochemical analysis that revealed the strongest signal upon concomitant TGF-β and *sox9* gene transfer at the highest vector doses applied (20 μl each vector) relative to rAAV-*lacZ* and to untreated aspirates (Fig. 1a). These results was corroborated by the results of an TGF-β ELISA (up to 1.9-fold higher amounts of TGF-β produced when co-applying the TGF-β and *sox9* vectors at the highest vector doses *versus* control conditions, *p* ≤ 0.050) (Fig. 1b). Strongest *sox9* expression was also observed in aspirates modified by rAAV-FLAG-h*sox9*/rAAV-hTGF-β at the highest vector doses while expression of the FLAG tag was only detected when applying the *sox9* vector (Fig. 1a).

**Table 1:** Evaluation of the % of β-gal^+^ cells in rAAV-transduced human bone marrow aspirates (day 21)

Condition	% of β-gal^+^ cells
no vector	21.6 (0.8)
*lacZ* (20 μl)	54.5 (2.8)^a,b^
*lacZ* (40 μl)	75.1 (1.9)^a,b^
TGF-β + *sox9* (10 μl + 10 μl)	20.9 (1.8)
TGF-β + *sox9* (20 μl + 20 μl)	20.3 (2.1)

Data are given as mean (SD). Statistically significant compared with ^a^(TGF-β + *sox9*) and ^b^(no vector) conditions (*p* ≤ 0.010)

### Activation of the proliferative, biosynthetic, and chondrogenic activities in human bone marrow aspirates upon co-overexpression of TGF-β and *sox9* via rAAV vectors

We next examined whether rAAV-mediated TGF-β and *sox9* co-overexpression was capable of modulating the proliferative, metabolic, and differentiation activities in human bone marrow aspirates maintained over time under chondrogenic stimulation relative to control conditions (rAAV-*lacZ*, no vector treatment).

**Fig. 2:**
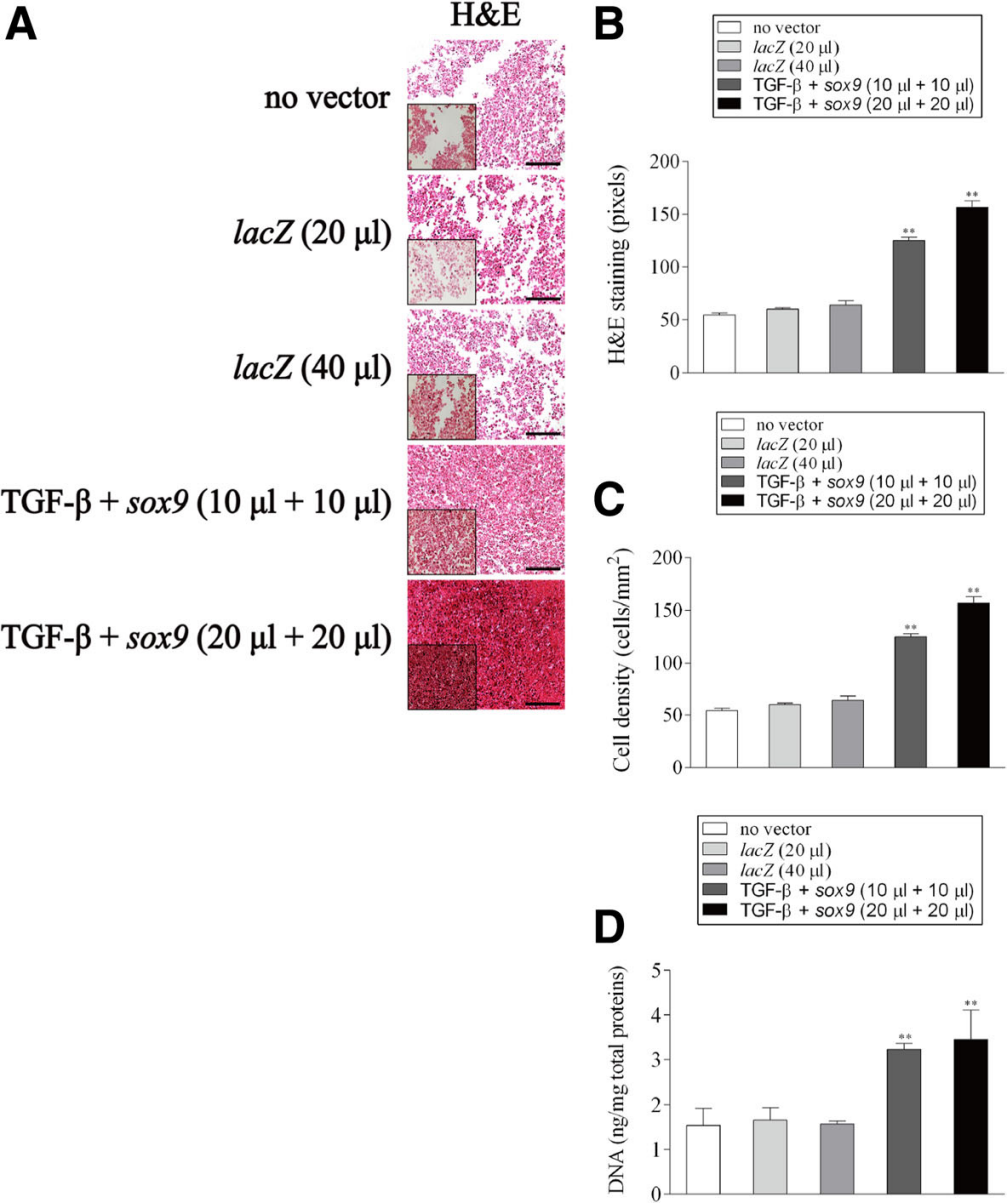
Proliferative activities in human bone marrow aspirates co-transduced with rAAV-hTGF-β/rAAV-FLAG-h*sox9*. Aspirates were treated and maintained in culture as described in Fig. 1. The samples were processed after 21 days to monitor the levels of cell proliferation by (**a**) H&E staining (original magnification x20, bar = 100 μm, insets at x40, all representative data) with histomorphometric analyses of the intensities of H&E staining (**b**) and of the cell densities (**c**), and (**d**) by estimating the DNA contents in the aspirates as described in the Materials and Methods. *,**Statistically significant compared with control groups (rAAV-*lacZ*, no vector condition) (*p ≤ 0.050, **p ≤ 0.010)

**Fig. 3:**
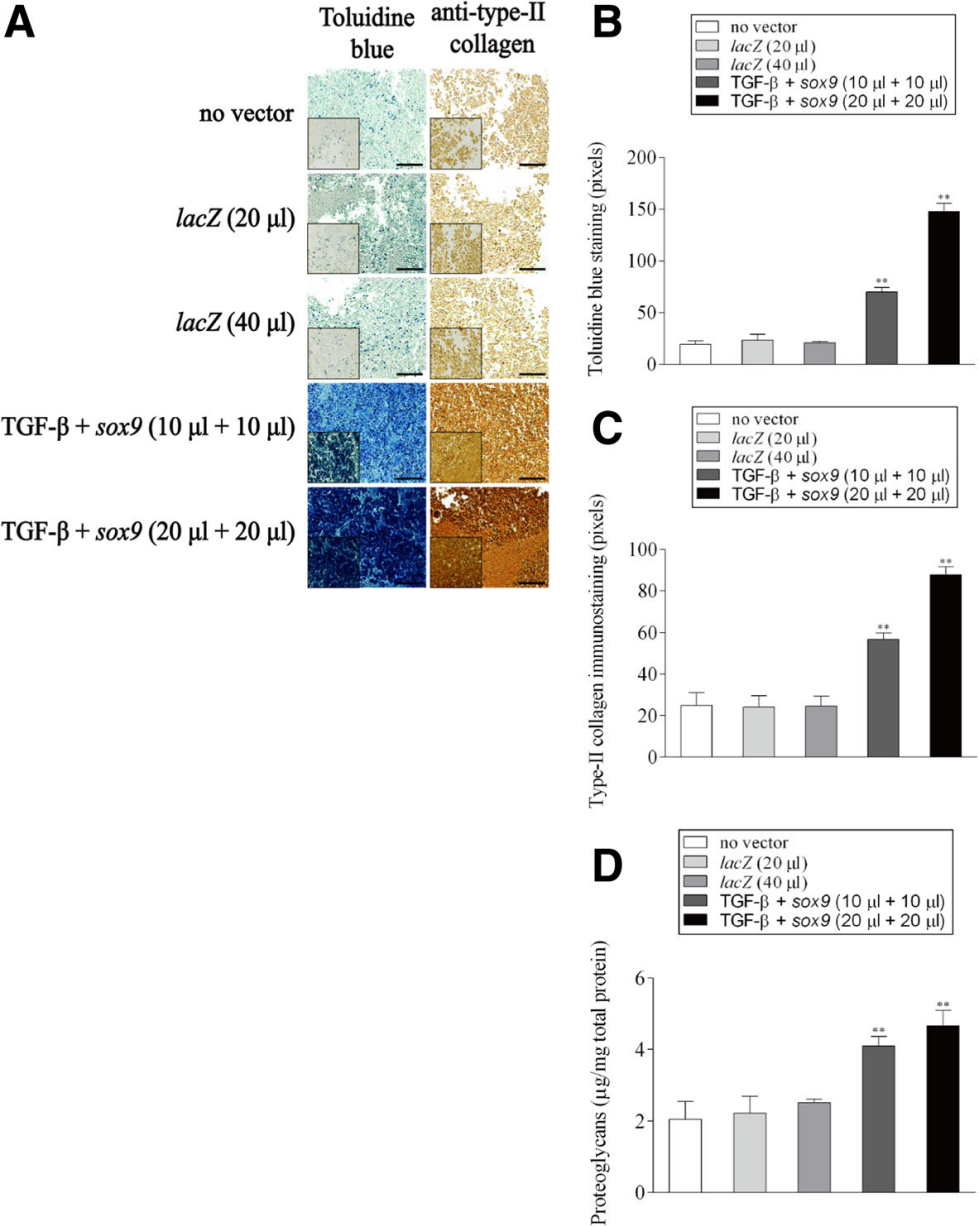
Biosynthetic activities and chondrogenic differentiation processes in human bone marrow aspirates co-transduced with rAAV-hTGF-β/rAAV-FLAG-h*sox9*. Aspirates were treated and maintained in culture as described in Figs. 1 and 2. The samples were processed after 21 days to evaluate the production of (**a**) matrix proteoglycans (toluidine *blue staining*) and type-II collagen (immunostaining) (original magnification x20, bar = 100 μm, insets at x40, all representative data) with histomorphometric analyses of the intensities of toluidine blue staining (**b**) and of type-II collagen immunostaining (**c**), and (**d**) by estimating the proteoglycan contents in the aspirates as described in the Materials and Methods. *,**Statistically significant compared with control groups (rAAV-*lacZ*, no vector condition) (*p ≤ 0.050, **p ≤ 0.010)

High, significant levels of proliferation were noted when co-delivering the TGF-β and *sox9* vectors to the aspirates for 21 days especially at the highest vector doses compared with the other conditions as noted by histomorphometric evaluations performed on H&E-stained sections from aspirates (up to 3.2-fold difference for both the H&E staining intensities and cell densities, *p* ≤ 0.010) (Fig. 2a-c), a finding corroborated when estimating the DNA contents in the samples (up to 2.7-fold difference, *p* ≤ 0.010) (Fig. 2d). Elevated, significant levels of matrix synthesis and chondrogenic differentiation were also achieved upon TGF-β and *sox9* co-gene transfer in the aspirates after 21 days particularly at the highest vector doses relative to the other conditions as observed by histomorphometric evaluations performed on histological sections from aspirates stained for toluidine blue and type-II collagen (up to 7.5- and 3.8-fold difference for the toluidine blue staining intensities and for those of type-II collagen immunostaining, respectively, *p* ≤ 0.010) (Fig. 3a-c), a finding supported by the results of an estimation of the proteoglycan contents in the samples (up to 2.5-fold difference, *p* ≤ 0.010) (Fig. 3d). Overall, these findings were corroborated by the results of a real-time RT-PCR analysis revealing enhanced levels of chondrogenic SOX9, ACAN, and COL2A1 expression upon concomitant TGF-β and *sox9* gene delivery in the aspirates after 21 days particularly when providing vectors at the highest vector doses compared with the other conditions (up to 8.9-, 30-, and 2.6-fold difference for SOX9, ACAN, and COL2A1, respectively, *p* ≤ 0.010) (see Fig. 5).

### Effects of rAAV-mediated TGF-β and *sox9* co-overexpression on the hypertrophic and terminal differentiation processes in human bone marrow aspirates

We finally evaluated the potential effects of TGF-β and *sox9* co-overexpression via rAAV on the occurrence of hypertrophic events in chondrogenically induced human bone marrow aspirates over time compared with control conditions (rAAV-*lacZ*, no vector treatment).

**Fig. 4:**
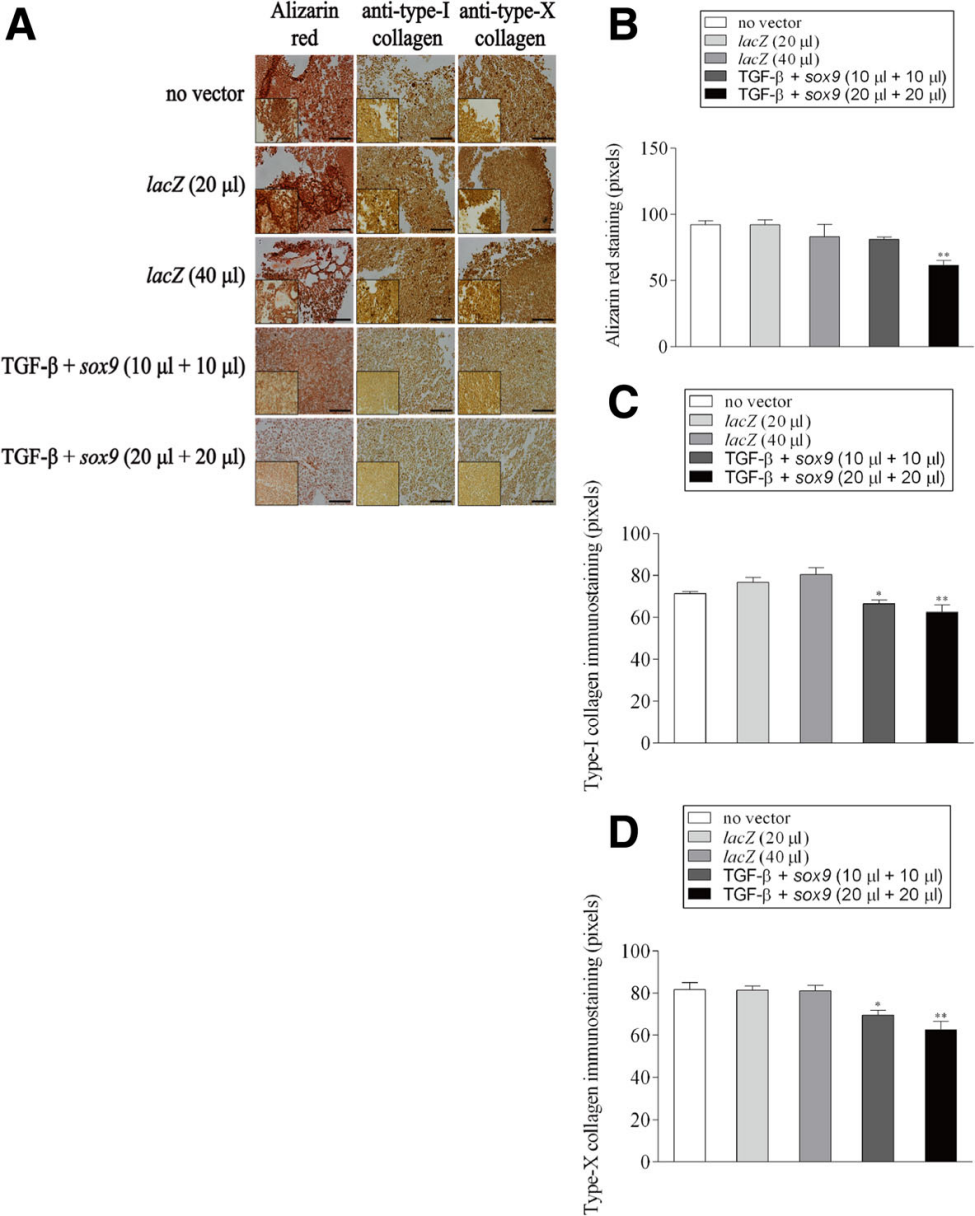
Hypertrophic and terminal differentiation processes in human bone marrow aspirates co-transduced with rAAV-hTGF-β/rAAV-FLAG-h*sox9*. Aspirates were treated and maintained in culture as described in Figs. 1, 2 and 3. The samples were processed after 21 days to evaluate (**a**) matrix mineralization (alizarin *red staining*) and the production of type-I and -X collagen (immunostaining) (original magnification x20, bar = 100 μm, insets at x40, all representative data) with histomorphometric analyses of the intensities of alizarin red staining (**b**) and of type-I collagen (**c**) and type-X collagen immunostaining (**d**) as described in the Materials and Methods. *,**Statistically significant compared with the control groups (rAAV-*lacZ*, no vector condition) (*p ≤ 0.050, **p ≤ 0.010)

**Fig. 5:**
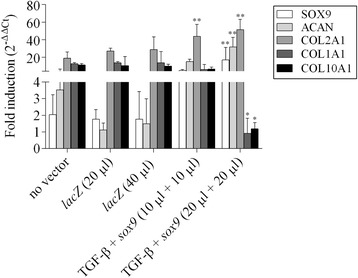
Expression analyses via real-time RT-PCR in human bone marrow aspirates co-transduced with rAAV-hTGF-β/rAAV-FLAG-h*sox9*. Aspirates were treated and maintained in culture as described in Figs. 1, 2, 3 and 4. Total cellular RNA extraction and cDNA synthesis with subsequent analysis of gene expression via real-time RT-PCR amplification was performed after 21 days as described in the Materials and Methods to generate Ct values for each target. The transcription factor SOX9, aggrecan (ACAN), type-II collagen (COL2A1), type-I collagen (COL1A1), and type-X collagen (COL10A1) were analyzed, with GAPDH serving as a housekeeping gene and internal control for normalization (primers are listed in the Materials and Methods). Fold inductions were measured for each target by using the 2^-ΔΔCt^ method relative to the control groups (rAAV-*lacZ*, no vector condition). *,**Statistically significant compared with control groups (rAAV-*lacZ*, no vector condition) (*p ≤ 0.050, **p ≤ 0.010)

Remarkably, co-application of rAAV-hTGF-β/rAAV-FLAG-h*sox9* at the highest vector doses reduced the levels of hypertrophy and terminal differentiation in the aspirates after 21 days compared with the other conditions as noted by histomorphometric evaluations performed on histological sections from aspirates stained with alizarin red, type-I, and -X collagen (up to 1.5-, 1.2-, and 1.3-fold difference for the alizarin red staining intensities and for those of type-I and -X collagen immunostaining, respectively, *p* ≤ 0.010) (Fig. 4a-d). These results were again supported by findings of a real-time RT-PCR analysis, showing lower levels of COL1A1 and COL10A1 expression using TGF-β and *sox9* at the highest vector doses after 21 days relative to the other conditions (up to 15- and 11.2-fold difference for COL1A1 and COL10A1, respectively, *p* ≤ 0.050) (Fig. 5).

## Discussion

As a novel, single-step approach to treat articular cartilage lesions, direct transplantation of bone marrow concentrates has the advantage of eliminating the complex and invasive preparation and expansion of progenitor cells to treat articular cartilage lesions (Johnstone et al. 2013; Orth et al. 2014). Yet, while already used in the clinics, such a procedure has not allowed to reproduce a functional hyaline cartilage in patients and instead the repair tissue formed in treated lesions remains of low quality (Gigante et al. 2012; Kim et al. 2014; Slynarski et al. 2006). Chondrogenic modification of such aspirates especially using the clinically adapted rAAV gene transfer vectors prior to re-implantation in the site of injury might be a potent tool to improve the processes of cartilage repair (Frisch et al. 2015). Here, we evaluated the potential benefits of concomitantly overexpressing the chondrogenic TGF-β and *sox9* factors using multiple rAAV vectors in human bone marrow aspirates with respect to their stimulatory effects as independent treatments in similar samples (Frisch et al. 2016; Rey-Rico et al. 2015) or as a combination in isolated human MSCs (Tao et al. 2016).

Our results first indicate that co-overexpression of TGF-β and *sox9* via rAAV can be successfully achieved at very high levels (~75% transduction efficiencies) in the aspirates over prolonged periods of time (at least 21 days), in good agreement with our previous observations in such samples (Rey-Rico et al. 2015). For comparison, other groups demonstrated the possibility of transducing rabbit and sheep aspirates with more immunogenic adenoviral vectors but with much higher vector doses (10^10^ adenoviral particles per 250 μl of aspirate *versus* 8 × 10^5^ rAAV particles per 100 μl here, i.e. a 5 × 10^3^-fold difference) (Ivkovic et al. 2010; Pascher et al. 2004). Of further note, combined TGF-β/*sox9* gene transfer (especially at the highest vector doses employed) allowed for the sustained expression of SOX9 as previously noted with single rAAV-FLAG-h*sox9* transduction (Rey-Rico et al. 2015) and to a durable production of TGF-β relative to the control conditions, in the range of those achieved when providing rAAV-hTGF-β alone (Frisch et al. 2016). While early on, all types of cells forming the aspirates (MSCs, hematopoietic cells, fibroblast-like cells) might be permissive to genetic modification, over time mostly MSCs may promote rAAV-mediated overexpression of the transgenes when continuously maintained under MSC-specific chondrogenic culture conditions (Frisch et al. 2016).

The present data further show that prolonged, effective co-overexpression of TGF-β and *sox9* led to increased levels of cell proliferation, matrix biosynthesis, and chondrogenic differentiation in the aspirates over time (at least 21 days), concordant with the properties of these factors (Babister et al. 2008; Bi et al. 1999; Cucchiarini et al. 2009; Frisch et al. 2014; Ikeda et al. 2004; Johnstone et al. 1998; Kim and Im 2011; Lee et al. 2011; Pagnotto et al. 2007; Steinert et al. 2009; Tao et al. 2016; Venkatesan et al. 2012) and with our previous work using independent gene transfer in similar samples (Frisch et al. 2016; Rey-Rico et al. 2015), and with effects in the range of those achieved when co-delivering these factors to isolated human MSCs (Tao et al. 2016). Again, such effects over time might be attributed to modified MSCs under continuous chondrogenic stimulation, while early on, other potentially transduced cell types may be active via paracrine effects (Frisch et al. 2016). Equally important, combined TGF-β/*sox9* transduction advantageously delayed premature hypertrophic differentiation in the aspirates *versus* control treatments, probably due to the effective, sustained overexpression of SOX9, a well-known anti-hypertrophic factor (Rey-Rico et al. 2015; Venkatesan et al. 2012) that may counterbalance possible hypertrophic effects of TGF-β (Barry et al. 2001; Frisch et al. 2014; Yoo et al. 1998).

Overall, we provide notable evidence of the potential of multiple gene-based approaches as a means to stimulate the chondroreparative activities in human bone marrow aspirates in order to generate practical, direct systems for implantation in sites of articular cartilage injury. Work is ongoing using samples from animal species (minipigs, sheep) in order to adapt the current strategy for administration in clinically relevant, orthotopic models of focal cartilage defects that provide the natural biochemical and biomechanical microenvironment for chondrogenesis (Cucchiarini et al. 2005; Ivkovic et al. 2010; Pascher et al. 2004). Such work may confirm the therapeutic activities of dual TGF-β/*sox9* gene transfer via rAAV and the advantageous delay in hypertrophic differentiation in vivo as mediated here by SOX9 in vitro (as discrepancies may occur in vivo) to counteract possible deleterious effects of TGF-β occurring upon direct gene transfer (osteophyte formation, fibrogenesis) (Bakker et al. 2001; Mi et al. 2003).

## Conclusion

The present study describes novel, convenient strategies via rAAV-mediated multiple gene transfer to modulate the chondrogenic differentiation processes in human bone marrow concentrates for the treatment of damaged articular cartilage. Gene transduction efficiencies, cell proliferation, matrix biosynthesis, and chondrogenic differentiation activities were systemically analyzed and compared over time in aspirates among all distinct subgroups, revealing that co-overexpression of TGF-β with SOX9 via rAAV effectively and durably enhanced such key chondroregenerative activities. Evaluation in relevant, preclinical models is ongoing, requesting to extensively translate the current procedure in animal samples, in order to determine the benefits (and monitor potential hurdles) of the approach in vivo prior to safe translation in the clinics. Additional work in human samples will be also further needed taking into account the impact of age, gender, and patient pathology on the bone marrow aspirate responses to the current treatment.

## Abbreviations

ACAN: Aggrecan
BMP: Bone morphogenetic protein
cDNA: Complementary deoxyribonucleic acid
CMV-IE: Cytomegalovirus immediate-early
COL10A1: Type-X collagen
COL1A1: Type-I collagen
COL2A1: Type-II collagen
COMP: Cartilage oligomeric matrix protein
Ct: Threshold cycle
DMEM: Dulbecco’s modified Eagle’s medium
ELISA: Enzyme-linked immunosorbent assay
FGF-2: Basic fibroblast growth factor
GAPDH: Glyceraldehyde-3-phosphate dehydrogenase
H&E: Hematoxylin and eosin
IGF-I: Insulin-like growth factor I
Ihh: Indian hedgehog
MSCs: Mesenchymal stem cells
PCR: Polymerase chain reaction
rAAV: Recombinant adeno-associated virus
RNA: Ribonucleic acid
RT-PCR: Reverse-transcriptase polymerase chain reaction
SD: Standard deviation
siRNA: Small interference RNA
SOX: Sex-determining region Y-type high mobility group box
TGF-β: Transformation growth factor beta
Wnt11: Wingless/Int 11
ZNF145: Zinc finger protein 145

## References

[CR1] Babister JC, Tare RS, Green DW, Inglis S, Mann S, Oreffo RO (2008). Genetic manipulation of human mesenchymal progenitors to promote chondrogenesis using “bead-in-bead” polysaccharide capsules. Biomaterials.

[CR2] Bakker AC, van de Loo FA, van Beuningen HM, Sime P, van Lent PL, van der Kraan PM, Richards CD, van den Berg WB (2001). Overexpression of active TGF-beta-1 in the murine knee joint: evidence for synovial layer-dependent chondro-osteophyte formation. Osteoarthritis Cartilage.

[CR3] Barry F, Boynton RA, Liu B, Murphy JM (2001). Chondrogenic differentiation of mesenchymal stem cells from bone marrow: differentiation-dependent gene expression of matrix components. Exp Cell Res.

[CR4] Bi WJ, Deng M, Zhang Z, Behringer RR, de Crombrugghe B (1999). Sox9 is required for cartilage formation. Nat Genet.

[CR5] Breinan HA, Martin SD, Hsu HP, Spector M (2000). Healing of canine articular cartilage defects treated with microfracture, a type-II collagen matrix, or cultured autologous chondrocytes. J Orthop Res.

[CR6] Cucchiarini M, Madry H, Ma C, Thurn T, Zurakowski D, Menger MD, Kohn D, Trippel SB, Terwilliger EF (2005). Improved tissue repair in articular cartilage defects in vivo by rAAV-mediated overexpression of human fibroblast growth factor 2. Mol Ther.

[CR7] Cucchiarini M, Terwilliger EF, Kohn D, Madry H (2009). Remodelling of human osteoarthritic cartilage by FGF-2, alone or combined with Sox9 via rAAV gene transfer. J Cell Mol Med.

[CR8] Cucchiarini M, Ekici M, Schetting S, Kohn D, Madry H (2011). Metabolic activities and chondrogenic differentiation of human mesenchymal stem cells following recombinant adeno-associated virus-mediated gene transfer and overexpression of fibroblast growth factor 2. Tissue Eng Part A.

[CR9] Frisbie DD, Oxford JT, Southwood L, Trotter GW, Rodkey WG, Steadman JR, Goodnight JL, McIlwraith CW (2003). Early events in cartilage repair after subchondral bone microfracture. Clin Orthop Relat Res.

[CR10] Frisch J, Venkatesan JK, Rey-Rico A, Schmitt G, Madry H, Cucchiarini M (2014). Determination of the chondrogenic differentiation processes in human bone marrow-derived mesenchymal stem cells genetically modified to overexpress transforming growth factor-β via recombinant adeno-associated viral vectors. Hum Gene Ther.

[CR11] Frisch J, Venkatesan JK, Rey-Rico A, Madry H, Cucchiarini M (2015). Current progress in stem cell-based gene therapy for articular cartilage repair. Curr Stem Cell Res Ther.

[CR12] Frisch J, Rey-Rico A, Venkatesan JK, Schmitt G, Madry H, Cucchiarini M (2016). TGF-β gene transfer and overexpression via rAAV vectors stimulates chondrogenic events in human bone marrow aspirates. J Cell Mol Med.

[CR13] Gigante A, Cecconi S, Calcagno S, Busilacchi A, Enea D (2012). Arthroscopic knee cartilage repair with covered microfracture and bone marrow concentrate. Arthrosc Tech.

[CR14] Haleem AM, Singergy AA, Sabry D, Atta HM, Rashed LA, Chu CR, El Shewy MT, Azzam A, Abdel Aziz MT (2010). The clinical use of human culture-expanded autologous bone marrow mesenchymal stem cells transplanted on platelet-rich fibrin glue in the treatment of articular cartilage defects: a pilot study and preliminary results. Cartilage.

[CR15] Haleem-Smith H, Calderon R, Song Y, Tuan RS, Chen FH (2012). Cartilage oligomeric matrix protein enhances matrix assembly during chondrogenesis of human mesenchymal stem cells. J Cell Biochem.

[CR16] Hauzeur JP, Gangji V (2010). Phases 1–3 clinical trials using adult stem cells in osteonecrosis and nonunion fractures. Stem Cells Int.

[CR17] Huang J, Zhao L, Xing L, Chen D (2010). MicroRNA-204 regulates Runx2 protein expression and mesenchymal progenitor cell differentiation. Stem Cells.

[CR18] Ikeda T, Kamekura S, Mabuchi A, Kou I, Seki S, Takato T, Nakamura K, Kawaguchi H, Ikegawa S, Chung UI (2004). The combination of SOX5, SOX6, and SOX9 (the SOX trio) provides signals sufficient for induction of permanent cartilage. Arthritis Rheum.

[CR19] Ivkovic A, Pascher A, Hudetz D, Maticic D, Jelic M, Dickinson S, Loparic M, Haspl M, Windhager R, Pecina M (2010). Articular cartilage repair by genetically modified bone marrow aspirate in sheep. Gene Ther.

[CR20] Jeon SY, Park JS, Yang HN, Woo DG, Park KH (2012). Co-delivery of SOX9 genes and anti-Cbfa-1 siRNA coated onto PLGA nanoparticles for chondrogenesis of human MSCs. Biomaterials.

[CR21] Johnstone BT, Hering M, Caplan AI, Goldberg VM, Yoo JU (1998). In vitro chondrogenesis of bone marrow-derived mesenchymal progenitor cells. Exp Cell Res.

[CR22] Johnstone B, Alini M, Cucchiarini M, Dodge GR, Eglin D, Guilak F, Madry H, Mata A, Mauck RL, Semino CE, Stoddart MJ (2013). Tissue engineering for articular cartilage repair--the state of the art. Eur Cell Mater.

[CR23] Kim HJ, Im GI (2011). Electroporation-mediated transfer of SOX trio genes (SOX-5, SOX-6, and SOX-9) to enhance the chondrogenesis of mesenchymal stem cells. Stem Cells Dev.

[CR24] Kim JD, Lee GW, Jung GH, Kim CK, Kim T, Park JH, Cha SS, You YB (2014). Clinical outcome of autologous bone marrow aspirates concentrate (BMAC) injection in degenerative arthritis of the knee. Eur J Orthop Surg Traumatol.

[CR25] Kon L, Filardo G, Roffi A, Di Martino A, Hamdan M, De Pasqual L, Merli ML, Marcacci M (2012). Bone regeneration with mesenchymal stem cells. Clin Cases Miner Bone Metab.

[CR26] Kuroda R, Ishida K, Matsumoto T, Akisue T, Fujioka H, Mizuno K, Ohgushi H, Wakitani S, Kurosaka M (2007). Treatment of a full-thickness articular cartilage defect in the femoral condyle of an athlete with autologous bone-marrow stromal cells. Osteoarthritis Cartilage.

[CR27] Lee HH, Haleem AM, Yao V, Li J, Xiao X, Chu CR (2011). Release of bioactive adeno-associated virus from fibrin scaffolds: effects of fibrin glue concentrations. Tissue Eng Part A.

[CR28] Liu TM, Guo XM, Tan HS, Hui JH, Lim B, Lee EH (2011). Zinc-finger protein 145, acting as an upstream regulator of SOX9, improves the differentiation potential of human mesenchymal stem cells for cartilage regeneration and repair. Arthritis Rheum.

[CR29] Liu TM, Ng WM, Tan HS, Vinitha D, Yang Z, Fan JB, Zou Y, Hui JH, Lee EH, Lim B (2013). Molecular basis of immortalization of human mesenchymal stem cells by combination of p53 knockdown and human telomerase reverse transcriptase overexpression. Stem Cells Dev.

[CR30] Liu S, Zhang E, Yang M, Lu L (2014). Overexpression of Wnt11 promotes chondrogenic differentiation of bone marrow-derived mesenchymal stem cells in synergism with TGF-β. Mol Cell Biochem.

[CR31] Liu P, Sun L, Chen H, Sun S, Zhou D, Pang B, Wang J (2015). Lentiviral-mediated multiple gene transfer to chondrocytes promotes chondrocyte differentiation and bone formation in rabbit bone marrow-derived mesenchymal stem cells. Oncol Rep.

[CR32] Madry H, van Dijk CN, Mueller-Gerbl M (2010). The basic science of the subchondral bone. Knee Surg Sports Traumatol Arthrosc.

[CR33] Mi Z, Ghivizzani SC, Lechman E, Glorioso JC, Evans CH, Robbins PD (2003). Adverse effects of adenovirus-mediated gene transfer of human transforming growth factor beta 1 into rabbit knees. Arthritis Res Ther.

[CR34] Nejadnik H, Hui JJ, Feng Choong EP, Tai BC, Lee EH (2010). Autologous bone marrow-derived mesenchymal stem cells versus autologous chondrocyte implantation: an observational cohort study. Am J Sports Med.

[CR35] Neumann AJ, Alini M, Archer CW, Stoddart MJ (2013). Chondrogenesis of human bone marrow-derived mesenchymal stem cells is modulated by complex mechanical stimulation and adenoviral-mediated overexpression of bone morphogenetic protein 2. Tissue Eng Part A.

[CR36] Orth P, Rey-Rico A, Venkatesan JK, Madry H, Cucchiarini M (2014). Current perspectives in stem cell research for knee cartilage repair. Stem Cells Cloning.

[CR37] Pagnotto MR, Wang Z, Karpie JC, Ferretti M, Xiao X, Chu CR (2007). Adeno-associated viral gene transfer of transforming growth factor-beta1 to human mesenchymal stem cells improves cartilage repair. Gene Ther.

[CR38] Pascher A, Palmer GD, Steinert A, Oligino T, Gouze E, Gouze JN, Betz O, SPector M, Robbins PD (2004). Gene delivery to cartilage defects using coagulated bone marrow aspirate. Gene Ther.

[CR39] Rey-Rico A, Frisch J, Venkatesan JK, Schmitt G, Madry H, Cucchiarini M (2015). Determination of effective rAAV-mediated gene transfer conditions to support chondrogenic differentiation processes in human primary bone marrow aspirates. Gene Ther.

[CR40] Samulski RJ, Chang S, Shenk T (1987). A recombinant plasmid from which an infectious adeno-associated virus genome can be excised in vitro and its use to study viral replication. J Virol.

[CR41] Samulski RJ, Chang S, Shenk T (1989). Helper-free stocks of recombinant adeno-associated viruses: normal integration does not require viral gene expression. J Virol.

[CR42] Skowronski J, Rutka M (2013). Osteochondral lesions of the knee reconstructed with mesenchymal stem cells - results. Ortop Traumatol Rehabil.

[CR43] Slynarski K, Deszczynski J, Karpinski J (2006). Fresh bone marrow and periosteum transplantation for cartilage defects of the knee. Transplant Proc.

[CR44] Steinert AF, Palmer GD, Pilapil C, Nöth U, Evans CH, Ghivizzani SC (2009). Enhanced in vitro chondrogenesis of primary mesenchymal stem cells by combined gene transfer. Tissue Eng Part A.

[CR45] Steinert AF, Weissenberger M, Kunz M, Gilbert F, Ghivizzani SC, Göbel S, Jakob F, Nöth U, Rudert M (2012). Indian hedgehog gene transfer is a chondrogenic inducer of human mesenchymal stem cells. Arthritis Res Ther.

[CR46] Tao K, Frisch J, Rey-Rico A, Venkatesan JK, Schmitt GT, Madry H, Lin J, Cucchiarini M (2016). Co-overexpression of TGF-β and SOX9 via rAAV gene transfer modulates the metabolic and chondrogenic activities of human bone marrow-derived mesenchymal stem cells. Stem Cell Res Ther.

[CR47] Venkatesan JK, Ekici M, Madry H, Schmitt G, Kohn D, Cucchiarini M (2012). SOX9 gene transfer via safe, stable, replication-defective recombinant adeno-associated virus vectors as a novel, powerful tool to enhance the chondrogenic potential of human mesenchymal stem cells. Stem Cell Res Ther.

[CR48] Wakitani S, Mitsuoka T, Nakamura N, Toritsuka Y, Nakamura Y, Horibe S (2004). Autologous bone marrow stromal cell transplantation for repair of full-thickness articular cartilage defects in human patellae: two case reports. Cell Transplant.

[CR49] Wakitani S, Nawata M, Tensho K, Okabe T, Machida H, Ohgushi H (2007). Repair of articular cartilage defects in the patello-femoral joint with autologous bone marrow mesenchymal cell transplantation: three case reports involving nine defects in five knees. J Tissue Eng Regen Med.

[CR50] Yoo JU, Barthel S, Nishimura K, Solchaga L, Caplan AI, Goldberg VM, Johnstone B (1998). The chondrogenic potential of human bone-marrow-derived mesenchymal progenitor cells. J Bone Joint Surg Am.

